# Intracellular Signaling by the *comRS* System in *Streptococcus mutans* Genetic Competence

**DOI:** 10.1128/mSphere.00444-18

**Published:** 2018-10-31

**Authors:** Simon A. M. Underhill, Robert C. Shields, Justin R. Kaspar, Momin Haider, Robert A. Burne, Stephen J. Hagen

**Affiliations:** a Department of Physics, University of Florida, Gainesville, Florida, USA; b Department of Oral Biology, University of Florida, Gainesville, Florida, USA; University of Iowa

**Keywords:** *Streptococcus mutans*, competence, microfluidics, quorum sensing

## Abstract

Entry into genetic competence in streptococci is controlled by ComX, an alternative sigma factor for genes that enable the import of exogenous DNA. In Streptococcus mutans, the immediate activator of *comX* is the ComRS quorum system. ComS is the precursor of XIP, a seven-residue peptide that is imported into the cell and interacts with the cytosolic receptor ComR to form a transcriptional activator for both *comX* and *comS*. Although intercellular quorum signaling by ComRS has been demonstrated, observations of bimodal expression of *comX* suggest that *comRS* may also function as an intracellular feedback loop, activating *comX* without export or detection of extracellular XIP. Here we used microfluidic and single-cell methods to test whether ComRS induction of *comX* requires extracellular XIP or ComS. We found that individual *comS*-overexpressing cells activate their own *comX*, independently of the rate at which their growth medium is replaced. However, in the absence of lysis they do not activate *comS*-deficient mutants growing in coculture. We also found that induction of *comR* and *comS* genes introduced into Escherichia coli cells leads to activation of a *comX* reporter. Therefore, ComRS control of *comX* does not require either the import or extracellular accumulation of ComS or XIP or specific processing of ComS to XIP. We also found that endogenously and exogenously produced ComS and XIP have inequivalent effects on *comX* activation. These data are fully consistent with identification of intracellular positive feedback in *comS* transcription as the origin of bimodal *comX* expression in S. mutans.

**IMPORTANCE** The ComRS system can function as a quorum sensing trigger for genetic competence in S. mutans. The signal peptide XIP, which is derived from the precursor ComS, enters the cell and interacts with the Rgg-type cytosolic receptor ComR to activate *comX*, which encodes the alternative sigma factor for the late competence genes. Previous studies have demonstrated intercellular signaling via ComRS, although release of the ComS or XIP peptide to the extracellular medium appears to require lysis of the producing cells. Here we tested the complementary hypothesis that ComRS can drive *comX* through a purely intracellular mechanism that does not depend on extracellular accumulation or import of ComS or XIP. By combining single-cell, coculture, and microfluidic approaches, we demonstrated that endogenously produced ComS can enable ComRS to activate *comX* without requiring processing, export, or import. These data provide insight into intracellular mechanisms that generate noise and heterogeneity in S. mutans competence.

## Abstract (precis)

The ComRS system can function as a quorum sensing trigger for genetic competence in S. mutans. The signal peptide XIP, which is derived from the precursor ComS, enters the cell and interacts with the Rgg-type cytosolic receptor ComR to activate *comX*, which encodes the alternative sigma factor for the late competence genes. Previous studies have demonstrated intercellular signaling via ComRS, although release of the ComS or XIP peptide to the extracellular medium appears to require lysis of the producing cells. Here we tested the complementary hypothesis that ComRS can drive *comX* through a purely intracellular mechanism that does not depend on extracellular accumulation or import of ComS or XIP. By combining single-cell, coculture, and microfluidic approaches, we demonstrated that endogenously produced ComS can enable ComRS to activate *comX* without requiring processing, export, or import. These data provide insight into intracellular mechanisms that generate noise and heterogeneity in S. mutans competence.

## INTRODUCTION

Streptococcus mutans inhabits human oral biofilms and is a primary etiological agent of dental caries (1). Many of the behaviors that facilitate the growth, competition, stress tolerance, and virulence of S. mutans are linked to the regulation of genetic competence, a transient physiological state during which the organism can internalize DNA from its environment (2–7). The competence pathway of S. mutans is complex, as it receives input from extracellular peptide signals and environmental cues as well as regulatory feedback (8–14). Consequently, several elements of the mechanism and dynamics of the pathway are not well understood.

S. mutans initiates entry into the competent state by increasing the transcription of the *comX* gene (sometimes referred to as *sigX*), which encodes an alternative sigma factor that is required for the expression of approximately 30 late competence genes (15, 16). Expression of *comX* can be induced by the peptides CSP (competence-stimulating peptide) and XIP (*sig* X-inducing peptide), and the efficacy of these peptides is strongly influenced by environmental conditions. CSP is derived by cleavage of a 21-residue peptide from ComC and exported through an ATP-binding cassette transporter. It is further processed to the active 18-residue peptide by the SepM protease (17). Extracellular CSP is detected by the two-component signal transduction system ComDE, with the phosphorylated response regulator ComE activating genes for bacteriocin synthesis and immunity. ComE does not directly activate *comX* but affects *comX* indirectly, via a pathway that is not completely understood (18).

The immediate regulator of *comX* in S. mutans and in streptococci of the salivarius, bovis, and pyogenic groups is the ComRS system. ComR is an Rgg-like cytosolic transcriptional regulator, and the type II ComS of S. mutans is a 17-residue peptide (19). The C terminus of ComS contains XIP, a 7-residue small hydrophobic peptide (SHP). Extracellular XIP is imported by the Opp permease and interacts with ComR to form a transcriptional activator for both *comX* and *comS* (19, 20). Notably, the S. mutans competence pathway contains at least two positive-feedback loops, as XIP/ComR activates *comS* and ComX activates *comE* expression (19, 20).

An intriguing property of S. mutans competence is that although exogenous CSP and XIP can both activate *comX* and induce transformability, they do so under different environmental conditions and elicit qualitatively different behaviors in the expression of *comX* (11). Exogenous CSP elicits a bimodal response in which less than half of the population activates *comX*, whereas exogenous XIP elicits a unimodal response in which all cells in the population activate *comX*. Further, CSP activates *comX* only in complex media containing small peptides; this activation requires that cells carry an intact *comS* but does not require the Opp permease (11). By contrast, exogenous XIP activates *comX* only in defined media lacking small peptides (11, 21); this activation requires that cells carry the *opp* gene but does not require *comS* (19). Therefore, although exogenous XIP can activate *comX* in a strain lacking *comS*, the bimodal *comX* response to CSP requires an intact *comS* gene.

The observation that competence in several streptococcal species is directly stimulated by an extracellular ComS-derived peptide suggests that ComRS constitutes a novel type of Gram-positive quorum signaling system, in which the ComS-derived XIP signal is processed and secreted, accumulates in the extracellular medium, and is then reimported. This interpretation in S. mutans is supported by several experimental observations. First, cells that carry *opp* take up exogenous XIP (in defined medium) and activate *comX* with high efficiency (19, 21). Second, exogenous synthetic XIP is dramatically more effective in stimulating transformability than is exogenous full-length ComS (19). Third, filtrates of S. mutans cultures grown to an optical density at 550 nm (OD_550_) of 0.4 in defined medium were able to stimulate a P*comX* reporter strain (21). Moreover, liquid chromatography-tandem mass spectrometry (LC-MS/MS) analysis of supernatants of S. mutans cultures grown to high density (22, 23) detected micromolar quantities of XIP. Fourth, a transposon mutagenesis screen in S. pyogenes identified the widely conserved *pptAB* ABC transporter as a possible exporter of short hydrophobic peptides of the ComS type (24), raising the possibility that S. mutans may also possess dedicated mechanisms for processing and export of ComS/XIP.

However, such mechanisms have not yet been identified in S. mutans. There is evidence that the Eep membrane protease facilitates the processing of S. thermophilus ComS (25), but Eep did not affect processing of ComS of S. mutans (22). Further, although S. mutans appears to encode a gene product with a fairly high degree of homology to PptAB of S. pyogenes, deletion of the apparent *pptAB* genes had only a weak effect on competence induction in mid-exponential-phase cultures of S. mutans (24). Consequently, the processing of ComS to XIP remains uncharacterized. The import of XIP presents an additional puzzle for ComRS quorum signaling because the permease Opp is required for XIP to activate *comX* but is not required for activation by CSP.

We recently demonstrated that XIP can function as a diffusible, intercellular signal (26) in S. mutans. A ComS-producing strain was able to induce *comX* response in cocultured cells lacking *comS*, in the absence of physical contact. However, intercellular signaling was greatly impaired by deletion of the *atlA* gene encoding the primary autolysin. Therefore, lysis and not active transport appears to be the primary route by which S. mutans externalizes the diffusible signal.

Here we used a combination of microfluidic and coculture methods to test the complementary hypothesis that ComRS can also activate *comX* by an intracellular mechanism, through endogenous ComS production, and that this mechanism does not specifically require processing of ComS/XIP or its extracellular accumulation.

## RESULTS

### An intact copy of *comS* alters the *comX* response to exogenous XIP.

**FIG 1:**
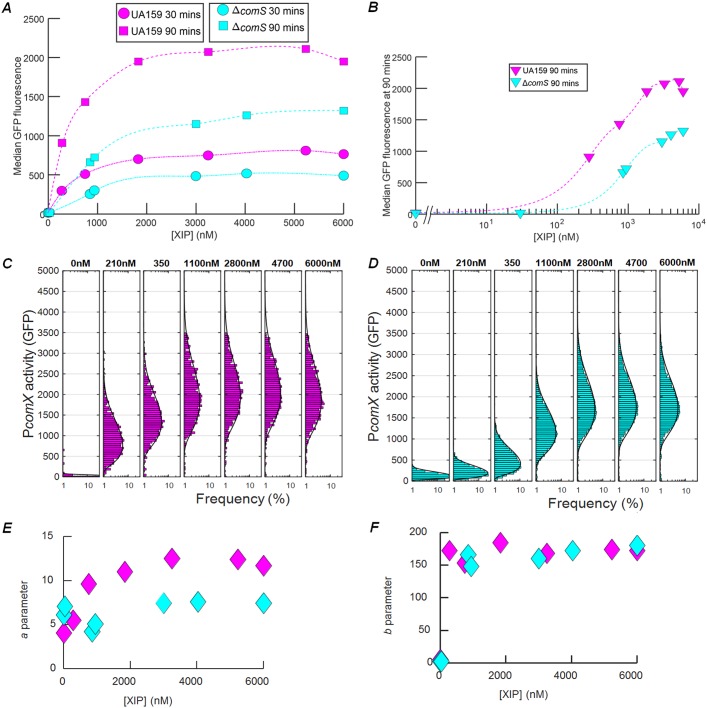
*comS* deletion is not fully complemented by synthetic XIP. (A) Comparison of levels of P*comX-gfp* activity in S. mutans cells of the UA159 (wild-type) background (magenta) and Δ*comS* mutant background (cyan). The median GFP fluorescence is shown for cells that were supplied with a continuous flow of exogenous synthetic XIP in FMC medium in microfluidic chambers. Data are shown at 30 min (circles, dashed-dotted lines) and 90 min (squares, dashed lines) of flow. The smooth curves represent spline fits to the data. (B) Median GFP levels in the two strains at the 90-min time point of the flow experiment. (C and D) Also shown are the histograms of the individual cell P*comX-gfp* reporter activity versus exogenous XIP concentration for (C) the UA159 background and (D) the Δ*comS* background. Solid black curves in panels C and D show the best-fit gamma probability distribution for each histogram. A cutoff of 40 units of P*comX-gfp* fluorescence has been applied to exclude background autofluorescence. (E) Parameter *a* of the (two-parameter) gamma probability distribution, obtained from the fits in panels C and D, reflecting the ratio of transcription rate to protein degradation rate. (F) Distribution parameter *b*, reflecting the ratio of the rate of translation to the rate of mRNA degradation. In panels E and F, cyan indicates the Δ*comS* mutant and magenta indicates the UA159 background.

A previous study found that even when a *comS* deletion strain (mutant *ΔcomS*) and a wild-type (WT) background strain were supplied with high concentrations of synthetic XIP, the transformation efficiency of the *ΔcomS* strain was only half that of the wild type (19). Panels A and B of Fig. 1 show results from a similar experiment performed with individual cells carrying a P*comX-gfp* plasmid-borne reporter. S. mutans UA159 (wild-type strain) and mutant Δ*comS* genetic backgrounds were imaged while adhered within a microfluidic chamber and supplied with a constant flow of defined medium (FMC [see Materials and Methods]) containing synthetic XIP. Although both strains respond to exogenous XIP, the Δ*comS* strain consistently showed roughly 1.5-fold-lower P*comX* activity than the wild type, even at saturating XIP concentrations. The threshold for *comX* response occurred at a roughly 2-fold-lower XIP concentration in UA159 than in the Δ*comS* strain (Fig. 1B). Therefore, the deletion of *comS* elevated the threshold for a response to extracellular XIP and reduced the overall response at saturation.

The deletion of *comS* also affected cell-to-cell variability (noise) in *comX* expression. Panels C and D of Fig. 1 show that the histograms of reporter fluorescence differ in UA159 (wild-type strain) and mutant Δ*comS* cells. UA159 showed a generally broader (noisier) *comX* response than did mutant Δ*comS*. We quantified this difference by fitting the histograms to a gamma distribution Γ(*n | a*,*b*), a two-parameter probability distribution that can be used to model cell-to-cell variability in *n*, the copy number for a bacterial protein (27). In a simple physical model, parameter *a* of the gamma distribution is related to the number of mRNAs produced during the cell division time, while parameter *b* is related to the number of protein copies produced per mRNA transcript (28). As shown in Fig. 1E and F, the UA159 background had a roughly 2-fold-higher value for parameter *a* (transcription rate), while the values for parameter *b* (translation) were similar for the two strains. As this difference persisted even at XIP concentrations exceeding 1 μM, these data show that deletion of *comS* significantly affected *comX* expression, even when excess extracellular XIP was provided.

### Fluid replacement did not alter induction of *comX* in a strain overexpressing *comS*.

To test whether *comX* activation requires cells to import extracellular XIP from their environment, we examined the effect of fluid flow rate in cells that overexpressed *comS* from the strong P23 promoter on the plasmid pIB184 (26, 29) while adhered in a microfluidic flow chamber. Cells carrying the 184*comS* overexpression plasmid produce significantly higher levels of *comS* mRNA (see Fig. S1 in the supplemental material) and activate *comX* in defined medium lacking exogenous CSP or XIP (26). We anticipated that if cells were immobilized and supplied with a continuous flow of fresh medium, high flow rates would remove any XIP (or ComS) that was released, leading to diminished *comX* activity. In order to prevent XIP import via Opp, cells were supplied with a flow of complex medium (brain heart infusion [BHI] medium) lacking XIP or CSP. We loaded 184*comS* P*comX-rfp* cells, which carry both the *comS* overexpression plasmid and a plasmid-borne P*comX-rfp* reporter, into four microfluidic chambers using different flow rates. Chambers were supplied with fresh complex medium flowing at rates between 0.02 ml h^−1^ and 1 ml h^−1^. These flow rates were sufficient to completely replace the growth medium within each chamber in time intervals ranging from 6 s to 10 min. We also studied (i) a P*comX-rfp* reporter in a UA159 background (negative control) and (ii) a ComS*-*overproducing strain lacking a start codon (ATG point mutation to AAG) on its chromosomal *comS* gene (mutant 184*comS* P*comX-rfp* Δ*comS*).

**FIG 2:**
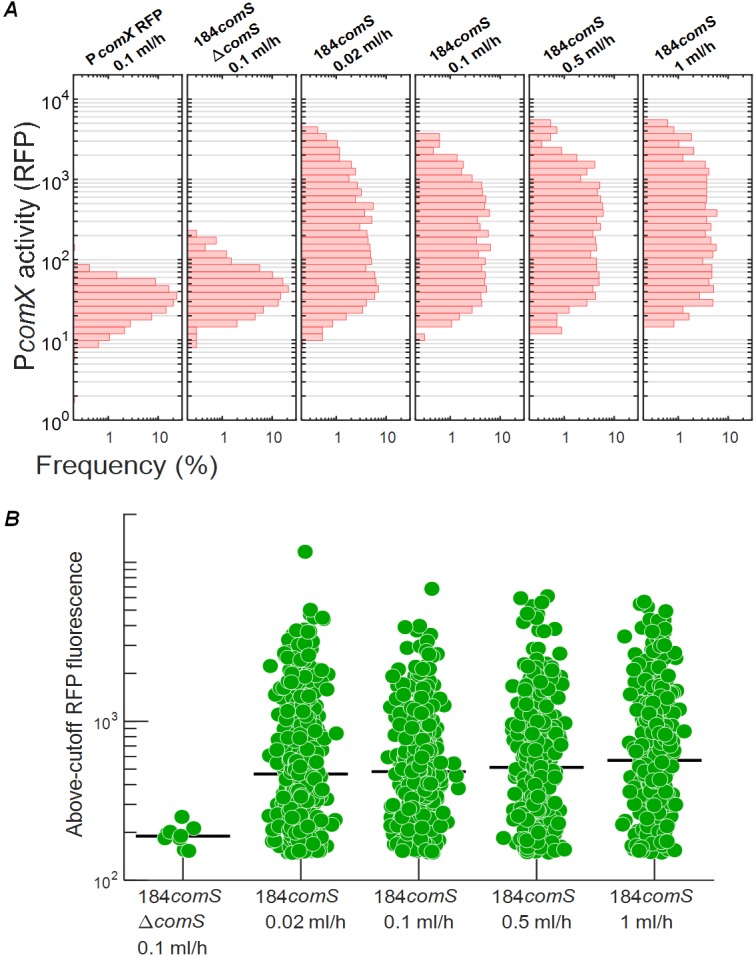
Activation of ComX in a ComS-overexpressing strain is independent of the rate of medium replacement. P*comX-rfp* reporter activity is shown in cells growing in microfluidic chambers supplied with continuously flowing fresh complex medium (BHI). (A) Histograms of individual cell P*comX-rfp* reporter fluorescence. (First column) wild-type background (negative control) with flow at 0.1 ml h*^−^*^1^. (Second column) ComS-overexpressing 184*comS* Δ*comS* background at 0.1 ml h*^−^*^1^. (Columns 3 to 5) ComS-overexpressing (184*comS*) background at 0.02 ml h*^−^*^1^, 0.1 ml h*^−^*^1^, 0.5 ml h*^−^*^1^, and 1 ml h*^−^*^1^. (At a flow rate of 1 ml h*^−^*^1^, the medium in each flow chamber is replaced every 6 s.) (B) RFP fluorescence of cells that exceeded the wild type (negative-control) red fluorescence in column 1 of panel A. The black bar indicates the median of data in each channel. (Leftmost column) 184*comS* Δ*comS* background. (Columns 2 to 5) ComS-overexpressing (184*comS*) background. All RFP measurements were made 4 h after addition of chloramphenicol to the cultures.

Figure 2A shows that P*comX* was not activated in the UA159 background (leftmost column). In contrast, the strain overexpressing *comS* and harboring an intact chromosomal copy of *comS* showed a highly heterogeneous response, indicating that a subpopulation of these cells strongly activated *comX* in the flowing complex medium. Further, the rate of fluid flow had no effect on their *comX* expression. Figure 2B shows the fluorescence of the individual cells for which the signal exceeded the maximum level of P*comX* activity (roughly 100 fluorescence units) seen in the UA159 negative control. Rather than declining at high flow rates where the medium was rapidly replaced, the median *comX* activity in the ComS*-*overproducing cells actually showed a very slight increase, smaller than the cell-to-cell variability. These data show that overexpression of *comS* can allow S. mutans to activate *comX*, even in complex medium which normally does not permit activation of *comX* transcription by exogenously supplied XIP. The finding that this activation is unaffected by rapid replacement of the medium implies that the *comX* response in the strain overexpressing *comS* was not due to accumulation of extracellular XIP (or ComS). Curiously, however, this *comX* response did require an intact chromosomal *comS* gene. Figure 2 shows that very few Δ*comS* cells activated *comX*, even though they harbored the *comS* overexpression plasmid. Together with Fig. 1, these data show that the chromosomal *comS* gene plays a role in *comX* activation that is not fully complemented either by saturating concentrations of exogenous XIP or by endogenous overproduction of ComS.

10.1128/mSphere.00444-18.3 FIG S1 Measurement of *comS*, *comX*, and *comR* transcript levels. (A to C) RT-qPCR measurement of (A) *comS*, (B) *comX*, and (C) *comR* transcripts in cultures harvested at an OD_600_ of 0.5. Each bar indicates the ratio of the median transcript count to the median 16S rRNA count, as measured in multiple biological and technical replicates (see Materials and Methods). WT/BHI, 184*comS*, and 184*comS* Δ*comS* samples were grown in BHI medium. The remaining samples were grown in FMC. Synthetic XIP was supplied at a 100 nM concentration when used. Error bars indicate the range from the second-lowest to the second-highest value among the replicates for each condition. (D) Comparison of *comS* mRNA transcripts from S. mutans harboring the ComS overexpression plasmid versus the empty plasmid. Download FIG S1, TIF file, 0.1 MB. Copyright © 2018 Underhill et al. 2018 Underhill et al. This content is distributed under the terms of the Creative Commons Attribution 4.0 International license.

### The presence of *comS* in the chromosome affects *comS* mRNA levels.

We used reverse-transcription quantitative PCR (RT-qPCR) to measure *comS*, *comR*, and *comX* transcript copy numbers in mid-exponential-phase cultures of the ComS overexpression, *comS* deletion, and UA159 strains (Fig. S1). Transcription of *comS* (Fig. S1A) was significantly increased in cells treated with exogenously added XIP in defined medium compared to controls lacking XIP or growing in complex medium. In addition, cells overexpressing *comS* in a wild-type genetic background (184*comS*) and growing in complex medium had *comS* transcript levels that were significantly higher than those seen with a strain overexpressing *comS* that lacked the chromosomal copy of *comS* (strain 184*comS* Δ*comS*). Levels of *comR* transcripts showed no significant change across strains or conditions (Fig. S1C).

### The population density of cells overexpressing *comS* does not determine the *comX* response.

To further test whether *comS-*overexpressing cells release extracellular XIP, we measured the effect of *comS*-overexpressing (sender) populations on *comX* activation in *comS*-deficient (receiver) cells growing in coculture. We mixed sender (184*comS* P*comX-rfp*) and receiver (P*comX-gfp* Δ*comS*) cultures in different ratios and loaded them into microfluidic chambers containing static medium without exogenous XIP. We anticipated that if the senders released XIP or ComS into the extracellular medium, both senders (red fluorescent protein [RFP] reporter) and receivers (green fluorescent protein [GFP] reporter) would respond by activating *comX* and that the average activation would increase with the ratio of senders to receivers. We used defined medium (FMC) to facilitate import of ComS/XIP if present.

**FIG 3:**
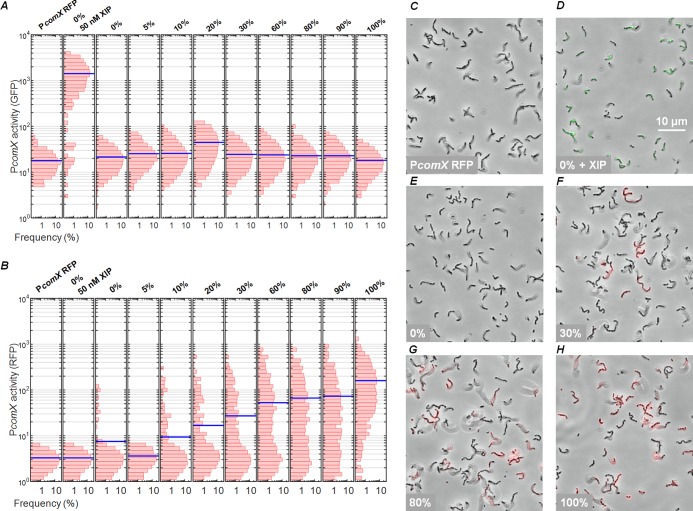
ComS overexpresser does not induce expression of *comX* of strain Δ*comS* in coculture. (A and B) Histograms of (A) GFP and (B) RFP fluorescence of individual cells in cocultures of sender (184*comS* P*comX-rfp*) and receiver (P*comX-gfp* Δ*comS*) strains in FMC medium. Strains were grown to equal levels of optical density, mixed in various proportions, and then incubated in microfluidic chambers containing stationary defined medium. Blue lines indicate population medians. Panels A and B show fluorescence of the UA159 background strain containing P*comX rfp* reporter, without added XIP (negative control) (first column); of strain P*comX-gfp* Δ*comS* (receiver) with added 50 nM XIP (positive control) (second column); and of cocultures of sender and receiver, with columns labeled by percentage by volume of 184*comS* (sender) culture in the initial preparation of the coculture. (C to H) Phase-contrast images of cocultures, overlaid with red and green fluorescence images. (C) P*comX-rfp* reporter in UA159 background, with no added XIP (negative control). (D) P*comX-gfp* Δ*comS* cells with 50 nM added XIP (positive control). (E) P*comX-gfp* Δ*comS* (receiver) alone, with 0% sender. (F to H) Cocultures containing 30%, 80%, and 100% sender, respectively.

We analyzed the green and red fluorescence of the cocultures to generate histograms of individual levels of cell fluorescence that revealed both the receiver (green) and sender (red) *comX* responses, shown in Fig. 3A and B, respectively. Representative microscopy images are shown in panels C to H of Fig. 3. In a control chamber containing only receiver cells exposed to 50 nM XIP, the GFP fluorescence was enhanced but RFP fluorescence remained at the baseline level (see Fig. 3D and the second column in Fig. 3A and B). Similarly, a control chamber containing only sender cells showed enhanced RFP fluorescence but only baseline GFP fluorescence (Fig. 3H and rightmost column in Fig. 3A and B). As expected, the median RFP fluorescence of the cocultures (Fig. 3B and F to H) increased at high sender/receiver ratios. The median RFP fluorescence of cells in the coculture increased approximately in proportion to the density of senders, as expected if each sender activated its own *comX*. RFP fluorescence was constant over a period of 4 h (Fig. S2).

10.1128/mSphere.00444-18.4 FIG S2 Response of cocultures is time independent. (A) GFP (*comX* reporter) and (B) RFP (*comY*) fluorescence of individual cells in cocultures of sender (184comS P*comX-rfp*) and receiver (P*comX-gfp* Δ*comS*) strains of S. mutans. Samples are labeled by percentage by volume of 184*comS* (sender) culture in the initial preparation of the coculture. Fluorescence was measured immediately (0 h, green) after mixing the coculture or 4 h (magenta) after mixing. The red horizontal bars show the median fluorescence immediately after mixing (0 h); the black vertical bars show the median at 4 h. Data are from the coculture experiment described in the Fig. 3 legend. Download FIG S2, TIF file, 0.2 MB. Copyright © 2018 Underhill et al. 2018 Underhill et al. This content is distributed under the terms of the Creative Commons Attribution 4.0 International license.

However, when exogenous XIP was not provided, the GFP fluorescence of cocultures showed no dependence on the sender/receiver ratio. Over the 0% to 100% range of coculture ratios shown in Fig. 3A, the GFP fluorescence histograms did not exceed the baseline level of the negative control (Δ*comS* strain alone) (see the third column of Fig. 3A). This GFP response did not change appreciably over a period of 4 h (Fig. S2). Senders did not activate *comX* in the Δ*comS* receivers at any mixing ratio.

These data show that although overexpression of *comS* stimulated *comX* within individual cells, over the 4-h time scale of this experiment, this activation did not cause extracellular, diffusible XIP to accumulate to levels sufficient to activate nearby Δ*comS* cells.

### Growth phase-dependent release of XIP.

**FIG 4:**
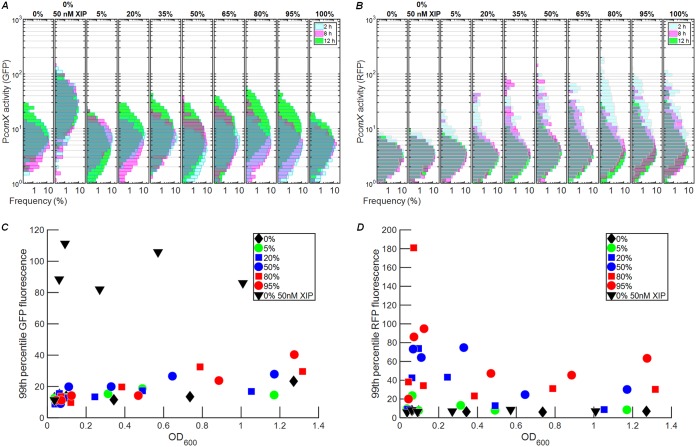
Evidence for release of XIP in cocultures late in growth. (A and B) Histograms of (A) GFP fluorescence and (B) RFP fluorescence of individual cells in cocultures of receiver (P*comX-gfp* Δ*comS*) and sender (P*comX-rfp* 184*comS*) strains in FMC, following different incubation periods. Labels at the top indicate the volume fraction comprised of the sender strain in the preparation of the coculture. Histogram colors indicate incubation times as follows: cyan, 2 h; magenta, 8 h; green, 12 h. The lower panels show the 99th percentile of the individual cell GFP fluorescence (C) and RFP fluorescence (D) observed in the culture, versus the culture OD_600_. Exogenous XIP was not added, except in the positive-control (sender-only) sample indicated by the inverted triangles in panels C and D.

We previously showed that intercellular signaling by S. mutans ComRS can occur late in growth but is impeded by deletion of the *atlA* gene, which encodes a major autolysin (26, 30). Loss of AtlA inhibits cell lysis, which appears to occur primarily in stationary phase. We therefore tested whether competence signaling from sender (*comS*-overexpressing) cells to receiver (Δ*comS*) cells would be observable in the later phases of growth. We prepared sender/receiver cocultures in different ratios in defined medium (to favor import of extracellular XIP if present). Because low pH suppresses the *comX* response, we adjusted the pH during the experiment to ensure that cells would remain responsive to *comX*-activating signals if present (9, 13). Every 2 h, the pH of the cultures was adjusted to 7.0 by addition of 2 N NaOH, the OD_600_ was recorded, and an aliquot of the culture was collected for fluorescence imaging of *comX* promoter activity. The GFP fluorescence histograms in Fig. 4A show that *comX* expression in the Δ*comS* strain increased very slightly at 12 h in comparison to expression at 2 h or 8 h. This increase was more pronounced at higher ratios of sender cells to receiver cells, consistent with some release of XIP from lysing senders late in the growth phase. The histograms of Fig. 4B, like those of Fig. 3B, show a strong RFP response (consistent with autoactivation of senders) with a slightly stronger response at earlier times.

The median GFP and RFP signals in the histograms described above do not shift dramatically with either time or coculture ratio. However, the histograms in Fig. 4A suggest moderate, density-dependent increases in receiver (green) fluorescence at 12 h. Panels C and D of Fig. 4 highlight these changes by showing the value of the 99th percentile of red and green fluorescence, respectively, in the cultures versus optical density. As shown in Fig. 4C, the GFP fluorescence of the most active receivers increased slightly at higher OD_600_ values, a change that was slightly more pronounced at higher sender/receiver ratios. By contrast, Fig. 4D shows no strong trend in the RFP fluorescence of the 99th percentile of senders versus OD_600_. None of the cocultures exhibited as strong a GFP response as the positive control (receiver plus 50 nM synthetic XIP; Fig. 4C), indicating that even 12 h of growth did not lead to an extracellular XIP accumulation of as large as 50 nM. Overall, these data are consistent with robust self-activation of the *comS* overexpressing strain, accompanied by a modest level of release of XIP (or ComS) to the extracellular medium during the late stages of the growth curve.

### Binding of ComR to the *comS* and *comX* promoters *in vitro*.

**FIG 5:**
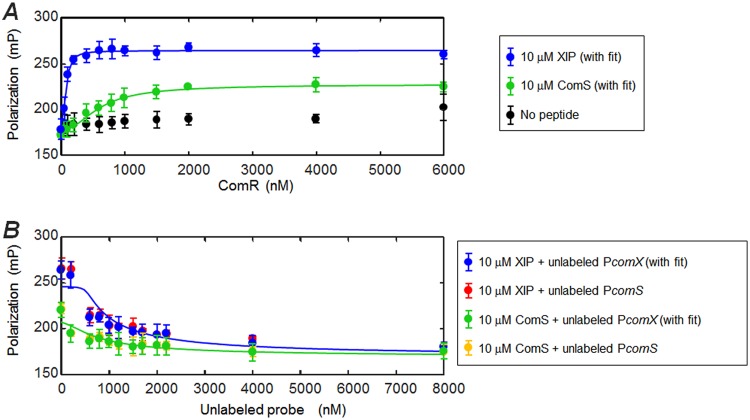
ComS and XIP interact with ComR to bind the *comX* promoter. A fluorescence polarization assay testing interaction of ComS and XIP peptides with ComR and the P*comX* transcriptional activation site was performed. The DNA probe was labeled with a Bodipy FL-X fluorophore. (A) Titration of ComR into a solution containing 10 µM XIP (blue) or full-length ComS (green), labeled DNA probe (1 nM), and 0.05 mg ml*^−^*^1^ salmon DNA. The negative control (black) contained no ComS or XIP peptide. (B) Competition assay in which unlabeled promoter sequence DNA was titrated into a solution containing ComR (1.5 µM), fluorescent DNA probe (1 nM), and peptide (either ComS or XIP, 10 µM). Unlabeled P*comX* DNA was used with XIP (blue) and ComS (green). An unlabeled P*comS* probe was also tested for its ability to compete with the fluorescent P*comX* probe in the presence of XIP (red) and ComS (gold). Solid curves indicate binding and competition behavior predicted by the two-step model described in Materials and Methods, in which peptide (ComS or XIP) first forms a multimeric complex with ComR (*k*_1_, *n*) and a single copy of this complex binds to the (labeled or unlabeled) P*comX* DNA (*k*_2_). For ComS binding/competition (green), the curves represent the following values: *k*_1_ = 3.2 μM, *k*_2_ = 2.2 nM, *n =* 2.5. For XIP binding/competition (blue), the curves represent the following values: *k*_1_ = 7.3 μM, *k*_2_ = 33 nM, *n =* 2.4.

The observation of *comX* activation in the overexpressing (sender) strain, without significant accumulation of XIP in the medium, implies that activation of *comX* does not require export and reimport of XIP or ComS if the chromosomal copy of *comS* is intact. To test whether endogenously produced ComS, acting intracellularly, is sufficient to activate *comX*, we tested whether unprocessed ComS could enable the binding of ComR to the *comS* and *comX* promoter regions *in vitro*. Figure 5A shows a fluorescence polarization (FP) assay with purified recombinant ComR, synthetic XIP or ComS, and a fluorescently labeled DNA oligomer corresponding to the S. mutans *comX* promoter region containing the ComR binding site. The assay was performed in the presence and absence of excess (10 µM) ComS or XIP. Because of the excess of peptide (10 μM) relative to fluorescent DNA probe (1 nM), the probe polarization depends primarily on the concentration of ComR added. Figure 5A shows that in the absence of XIP or ComS, ComR caused a weak rise in the fluorescence polarization of the DNA oligomer, indicating relatively weak affinity, as observed for ComR of other streptococci (20). However, in the presence of ComS or XIP the binding isotherm saturated at lower ComR concentrations, indicating formation of a complex with higher affinity for the *comX* promoter. Histidine tagging of ComR was found to reduce this affinity, as shown in Fig. S3.

10.1128/mSphere.00444-18.5 FIG S3 Effect of histidine tag on ComR binding of ComS and XIP. A fluorescence polarization study of ComS and XIP interaction with ComR that was N- or C-terminally tagged with 6×-histidine was performed. Polarization is plotted versus [ComR] for (A) C-terminally tagged ComR and (B) N-terminally tagged ComR. In each case, 1 nM fluorescent DNA and 0.05 mg ml^−1^ salmon DNA were present along with no signal peptides (black), XIP (blue), or ComS (green). The *comR* gene (S. mutans 61 [SMu.61]) was amplified using gene-specific primers (forward, AAAGAATCCTATGTTAAAAGA; reverse, CACCCTAGGAGACCCATCAAA) and cloned into the pET45b(+) vector bearing an N-terminal 6×His tag. The resulting vector, pET45b(+)his-comR_UA159_, was transformed into E. coli 10-beta. After sequencing confirmed the correct insertion (using T7 promoter and T7 terminator primers), the vector was transformed into E. coli BL21(DE3) prior to protein purification. Download FIG S3, TIF file, 0.1 MB. Copyright © 2018 Underhill et al. 2018 Underhill et al. This content is distributed under the terms of the Creative Commons Attribution 4.0 International license.

Figure 5B shows results of a competition assay in which unlabeled (“cold”) P*comX* and P*comS* DNA oligomers having the same stem-loop structure as the labeled probe were titrated into samples containing 1.5 µM ComR or 10 µM ComS or XIP and the labeled DNA (1 nM). The systematic decrease in polarization is consistent with competition for ComR. The unlabeled P*comS* and P*comX* probes, which differ by 3 bases, appear to have identical levels of affinity for ComR.

Figure 5A confirms a ComR-dependent interaction between ComS and the DNA probe, although this interaction is weaker than that of XIP. It also shows that, at saturating concentrations of ComR, ComS elicits only half of the total fluorescence polarization that results from an equivalent concentration of XIP. This difference may suggest that ComS and XIP induce qualitatively different interactions between the ComR/peptide complexes and the DNA probe. The solid curves in panels A and B of Fig. 5 were calculated from a two-step, cooperative binding model (see Materials and Methods) in which the dissociation constants for the ComR/peptide complex (*k*_1_) and the complex/promoter (*k*_2_), as well as the order of multimerization *n* of the ComR/peptide complex, are variables. Although the data clearly indicate that both ComS and XIP interact with ComR to bind the DNA probe, they do not permit a precise determination of the ComS and XIP interaction parameters. As discussed in Materials and Methods, the data are consistent with a range of parameter values, corresponding roughly to micromolar *k*_1_ and nanomolar *k*_2_ for both ComS and XIP and to similar cooperativity *n* values for the two peptides. The curves in Fig. 5 show the model with a roughly 2-fold difference in the ComR dissociation constants for ComS (*k*_1_ = 3.2 μM, *n =* 2.5) and XIP (*k*_1_ = 7.3 μM, *n =* 2.4).

We note that the binding observed with the ComS peptide is likely not attributable to a XIP impurity in the ComS, as ComS and XIP were used at levels of high excess and so a XIP impurity would have caused the blue and green curves in Fig. 5 to be much more qualitatively and quantitatively similar.

### ComRS can drive expression from the *comX* promoter in Escherichia coli.

**FIG 6:**
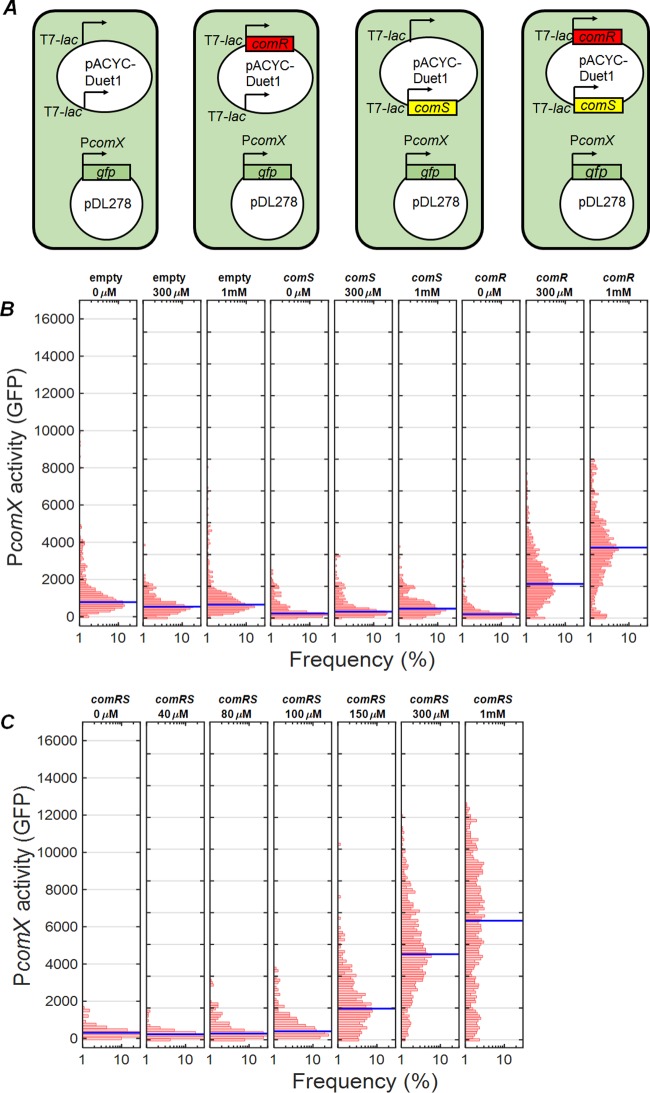
Activation of P*comX* by inducible *comRS* in E. coli. Activity of a P*comX-gfp* reporter within individual *E. coli* cells was measured by fluorescence microscopy. (A) Strains harbored a two-plasmid system that included the P*comX-gfp* reporter as well as different combinations of *comR* and *comS* under the control of the T7-*lac* promoter. (B) GFP fluorescence of controls containing the pACYC-Duet1 empty vector, *comR* alone, or *comS* alone after 3 h of growth with shaking, following addition of 0, 300 µM, and 1 mM IPTG. (C) GFP fluorescence of the strain possessing both *comR* and *comS* after 3 h of growth with shaking in various *IPTG* concentrations. The blue bar represents the median cell fluorescence.

As discussed above, the OppA permease, which imports extracellular XIP, is not required for CSP-driven stimulation of ComRS and *comX*. In addition, the export of XIP is accomplished by the nonspecific mechanism of lysis. To test whether the ComRS system requires dedicated mechanisms for processing of ComS to XIP, we constructed a dual-plasmid system that placed IPTG (isopropyl-β-d-thiogalactopyranoside)-inducible *comS* and *comR* into E. coli cells carrying a P*comX-gfp* reporter. Figure 6 shows the GFP activity of E. coli BL21(DE3) cells possessing the pDL278 P*comX-gfp* fusion and a pACYC-Duet1 plasmid that either was (i) empty plasmid or else carried inducible (ii) *comS*, (iii) *comR*, or (iv) *comR* plus *comS* (Fig. 6A). Figure 6B shows GFP activity of the control strains (i to iii) in response to added IPTG. Following induction by IPTG, the *comS* and empty vector controls showed only baseline GFP expression (Fig. 6B). However, *comR* alone was able to elicit some expression from the *comX* reporter, consistent with the weak DNA binding seen at high ComR concentrations (Fig. 5). When both *comR* and *comS* were present (Fig. 6C), the median *comX* expression was roughly 2-fold greater than the level seen with ComR alone at 1 mM IPTG and was roughly 3-fold higher at 300 µM IPTG. These data show that endogenously produced, unprocessed ComS is sufficient to enhance ComR-induced expression from the *comX* promoter.

### Data are consistent with an intracellular feedback loop in *comS* transcription.

**FIG 7:**
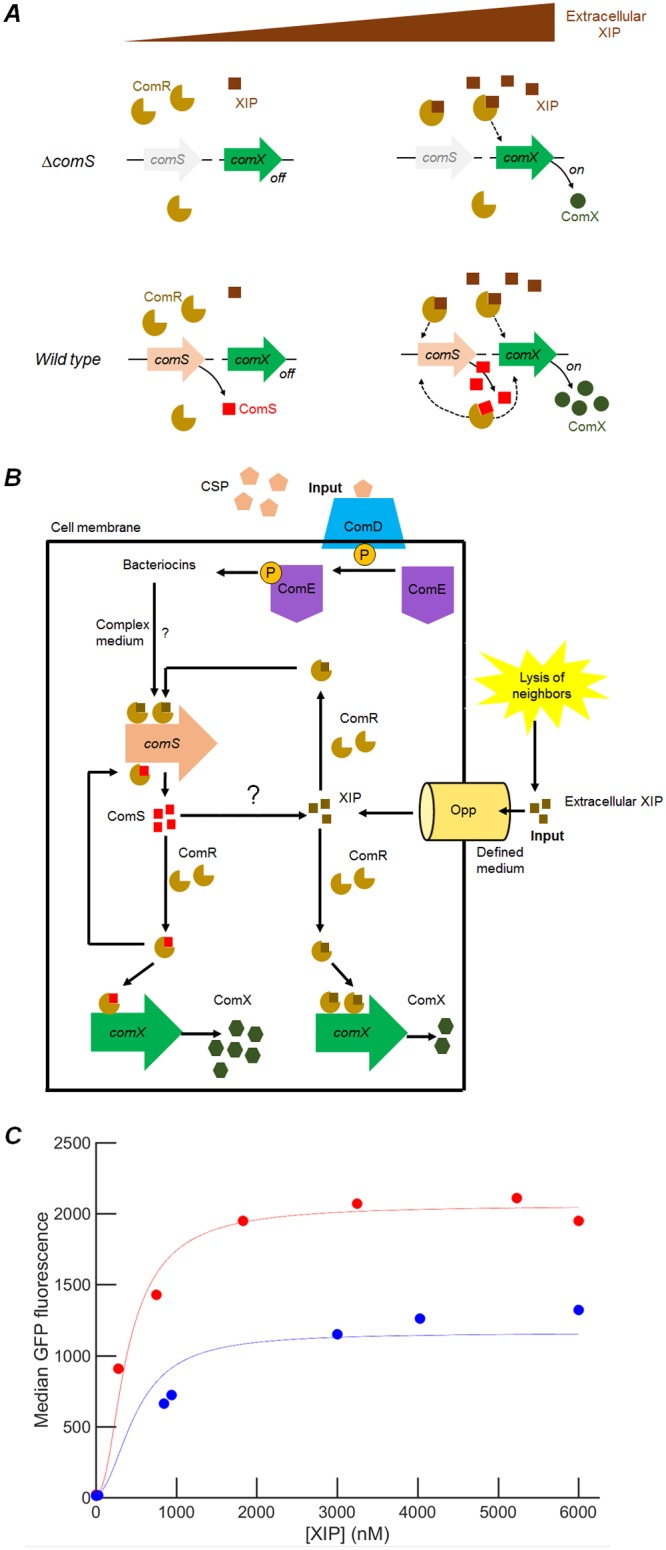
Proposed model for *comRS* feedback regulation of *comX*. The model represents *comS* feedback-enhanced activation of P*comX-gfp* reporter by exogenous synthetic XIP. (A) Illustration of the feedback model and the role of *comS* and extracellular XIP. ComS and XIP both interact with ComR to activate transcription of *comS* and *comX.* At low concentrations of extracellular XIP, *comS* expression is very low and *comX* is not expressed. At high concentrations of extracellular XIP, XIP is imported efficiently by Opp and interacts with ComR to drive expression of both *comS* and *comR*. Endogenously produced ComS is not readily exported in the absence of lysis, and so intracellular accumulation of ComS drives elevated *comS* and *comX* expression. Consequently, *comX* expression at any given XIP concentration is boosted by *comS* feedback. Cells lacking native *comS* can respond to synthetic XIP but cannot activate *comX* to the same level as the wild type. The figure presents the behavior seen in defined medium; in complex medium, extracellular XIP is not imported (11). (B) Integration of this intracellular feedback system into the wider *com* regulon, involving upstream activator ComE and its response to exogenous CSP through the ComD transmembrane kinase. ComS is allowed to be processed to XIP intracellularly by an unidentified protease. (C) Comparison of model fit with data. Red circles indicate median P*comX-gfp* fluorescence of the UA159 background strain supplied with synthetic XIP in microfluidic flow; blue circles indicate the median P*comX-gfp* fluorescence of the Δ*comS* background. Solid curves represent calculated values from a fit in which 11 parameters were fitted to the microfluidic data, as described in Materials and Methods. The model relates the predicted ComX concentration to the median GFP fluorescence by an offset and scale factor.

The reduced *comX* response in *comS* deletion strains in Fig. 1A and 2A shows that an intact *comS* gene has a stimulatory effect on *comX* expression that is not entirely matched either by exogenous XIP or by overexpression of ComS from a plasmid. One explanation for this effect is the presence of transcriptional positive feedback, in which endogenously produced ComS retained within the cell interacts with ComR to stimulate transcription from *comS* and *comX* (Fig. 7A). Import of extracellular XIP would be expected to stimulate such feedback in cells carrying an intact *comS* gene, potentially accounting for the stronger *comX* response to XIP that is observed in the wild-type strain versus the mutant Δ*comS* background. This intracellular system is integrated into the upstream CSP pathway through an unknown mechanism known to require bacteriocins, as indicated in Fig. 7B.

We constructed a mathematical model of this mechanism as described in the Materials and Methods and in Text S1 in the supplemental material. The model addresses conditions in a defined medium where extracellular XIP can be imported to the cell and early growth where lysis-driven release of ComS or XIP is not significant. Under these conditions, the model assumes that (i) extracellular XIP is imported to the cell but that (ii) neither ComS nor XIP is exported, though XIP may be intracellularly generated. (iii) Further, both the ComS and XIP complexes of ComR are able to elicit transcription of *comS* and *comX*, although not necessarily with equal transcription rates or degrees of multimerization. Assumption iii is motivated by data indicating that ComR binding does not absolutely require processing of ComS to XIP, by differences in the saturating fluorescence polarization achieved with ComS and XIP shown in Fig. 5A, and by the different saturating *comX* response (to XIP) of the Δ*comS* and UA159 backgrounds shown in Fig. 1A. In parametrizing the model, we allowed different orders of multimerization in the ComR/peptide complex that binds DNA: *n =* 1 (ComS) or *n =* 2 (XIP) (see Discussion). In addition, the maximal rate of transcription (ComX production) is permitted to depend on whether a ComR/XIP complex or a ComR/ComS complex is bound to the promoter.

10.1128/mSphere.00444-18.1 TEXT S1 Methods used for deterministic modeling of the ComRS activation of *comX* with intracellular feedback. Download Text S1, DOCX file, 0.01 MB. Copyright © 2018 Underhill et al. 2018 Underhill et al. This content is distributed under the terms of the Creative Commons Attribution 4.0 International license.

Figure 7C shows the results of solving the steady-state dynamical equations of the model with the parameter values that best reproduce the data (see Materials and Methods). If only XIP (but not full-length ComS) interacts with ComR to activate *comX*, the model gives identical levels of *comX* activation in both the Δ*comS* and wild-type backgrounds, in contradiction of the data presented in Fig. 1A. However, if both the ComR/XIP complexes and ComR/ComS complexes are permitted to induce transcription, although with different maximal rates, the model reproduces the different *comX* expression levels in the Δ*comS* and wild-type backgrounds. Parameter values (see Table S2 in the supplemental material) were found to preserve the relative orders of magnitude of the dissociation constants of the transcriptional activators found in Fig. 5, with the best fit giving a transcription rate that is between 4-fold and 200-fold greater for the ComS-ComR bound promoter than for XIP-ComR activation of the gene.

## DISCUSSION

The ComRS system found in mutans, salivarius, pyogenic, and bovis streptococci has been described alternatively as a quorum sensing mechanism (19) or timing mechanism (25) that directly controls *comX*, the master regulator of genetic competence. The ComS-derived XIP peptide is readily imported by S. mutans in defined growth medium, where it induces transformability with high efficiency. Key evidence supporting the idea of an intercellular signaling role for XIP includes the detection by LC-MS/MS of XIP in supernates of S. mutans cultures that were grown to high cell densities (22, 23). In addition, filtrates of S. mutans cultures grown to high density induced P*comX* activity in reporter strains (21), indicating the presence of an active competence signal in the extracellular medium. A recent coculture study verified that XIP is freely diffusible in aqueous media and that ComS-overexpressing senders are able to activate *comX* in nearby Δ*comS* receiver mutants, with no cell-cell contact being required (26). However, deletion of *atlA*, which encodes a surface-localized protein associated with envelope biogenesis and autolysis (30), suppressed this intercellular signaling (26). Taken together, these data indicate that the S. mutans ComRS system can provide intercellular competence signaling when autolysis releases sufficient concentrations of ComS or XIP.

We have previously argued that the bimodal expression of *comX* under conditions of stimulation by CSP is a signature indicating that *comX* is controlled by an intracellular transcriptional feedback system: endogenously produced ComS (or its product XIP) accumulates within some cells and then interacts with ComR to activate *comX.* Such feedback mechanisms are often a cause of bimodality in bacterial gene expression, including in other competence pathways (31, 32). The lack of an established mechanism for processing and export of ComS, the sensitivity of *comX* bimodality to endogenous production (via chromosomal *comS*) of XIP, and the fact that XIP import via Opp is not required for the bimodal behavior suggest together that purely intracellular signaling in *comRS* may be possible, at least under some environmental conditions. Such a mechanism would potentially allow individual S. mutans cells within a population to differ in competence behavior, as observed experimentally (14).

The present data support this model by providing several lines of evidence indicating that the chromosomal *comS* gene plays a role in ComRS activation of *comX*, regardless of the presence of other XIP or ComS that may be supplied. First, although the response of *comX* in complex medium supplemented with CSP is different from that seen in defined medium supplemented with XIP, the behavior of *comX* is affected in both cases by the presence of the intact chromosomal *comS* gene under the control of its cognate promoter. In complex medium, *comS* is required in order for CSP to elicit any *comX* response; in defined media, deletion of *comS* reduces the level of the *comX* response (both average and variance) to exogenous XIP and raises the threshold of the XIP concentration required for a response. Further, even if it harbors a *comS* overexpression plasmid, a *comS* deletion strain expresses *comX* much more weakly than does a *comS-*overexpressing strain that retains its chromosomal *comS* gene. These data show that the cell’s own native regulation of *comS* affects its activation of *comX*, independently of whether it overproduces ComS from a plasmid or imports exogenous XIP via Opp.

Our data also show that even in complex growth medium, which is known to inhibit the uptake of extracellular XIP, ComS-overexpressing (sender) cells activate their own *comX* gene. This autoactivation is unaffected by very rapid exchange (by flow) of the medium, strongly suggesting that *comX* activation in these cells does not require accumulation of extracellular XIP. However, this autoactivation requires the presence of a native *comS* gene in addition to the overexpression plasmid. In addition, even in the ComS overexpression strain, *comS* mRNA levels were significantly higher when the native *comS* was present, but *comR* mRNA levels were unaffected (see Fig. S1 in the supplemental material). These behaviors are consistent with the presence of positive feedback where imported XIP or plasmid-generated ComS enables internal amplification of *comS* expression.

Finally, the data show that *comS*-overexpressing cells fail to stimulate Δ*comS* cells (receivers) in cocultures under defined medium conditions, which are favorable for import of XIP. As the Δ*comS* receivers do respond to exogenously added XIP, these data indicate that the overexpressing cells activate their own *comX* without releasing significant XIP to the medium. The weak intercellular signaling that is observed in cocultures grown to late growth phases is consistent with eventual lysis of sender cells, possibly linked to autoactivation of the lytic pathway driven by ComX and ComDE.

The finding that diffusive signaling by ComS or XIP between S. mutans cells is inefficient or lacks spatial range is consistent with the conclusion reached by Gardan et al. using S. thermophilus (25). Those authors found that the type I ComS peptide of S. thermophilus was not secreted at detectable levels in a strain that produced it naturally, although an overproducing strain did generate detectable ComS in the medium. They argued that ComS does not diffuse through or accumulate in the medium, although it may be able to signal between cells that are in physical contact. This proximity model for ComS resembles a “self-sensing” quorum system (33) in which the secreted signal is retained at elevated concentrations in the immediate surroundings of the cell, possibly associated with the cell surface, so that the cell responds somewhat more strongly to its own secreted signal than to that of the rest of the population.

Our observations suggest more strongly that intercellular quorum signaling through ComS or XIP was not essential to ComRS control of *comX* in S. mutans under the conditions examined. A more essential component is the dynamics of the cell’s own *comS* transcription. A plausible mechanism for the bimodal response of S. mutans *comX* to CSP stimulation is therefore that CSP stimulates the bistable *comRS* feedback loop by facilitating, through an indirect mechanism, the positive feedback. It would be sufficient, for example, to inhibit degradation of endogenous ComS, which, depending on the basal transcription levels, would trigger *comX* activation in at least some cells, leading to the bimodal distribution of *comX* activity (11). Notably, our data show that overexpression of *comS* also leads to heterogeneous *comX* activity, suggesting that it plays a role similar to that played by exogenous CSP by facilitating *comS* autofeedback.

Since whether S. mutans ComS is processed to XIP inside the cell is unknown, either XIP or ComS could potentially act as the intracellular feedback signal. Although S. mutans competence was shown to be unresponsive to the presence of exogenous full-length ComS (19), this finding may reflect either selectivity by ComR or simply inefficient import of full-length ComS by Opp. ComS is significantly larger (17 residues) than the peptides that are typically transported by ABC transporters. Shanker et al. (34) found that S. mutans ComR is unresponsive to the ComS peptides produced by other streptococcal species, although an eight-residue XIP peptide (ComS_10-17_) did interact effectively with ComR to bind the *comS* and *comX* promoters (20). Our fluorescence polarization data confirm that both ComS and XIP can interact with ComR to bind the *comX* and *comS* promoter regions. They also suggest that ComS and XIP may form ComR complexes of different degrees of multimerization, a difference that could have interesting consequences for the nonlinear dynamics of feedback regulation. Our mathematical model for transcriptional autofeedback in the *comRS* system incorporates the data by assuming that endogenously produced ComS is not released to the environment, although extracellular XIP is imported and supplements the endogenous ComS in interacting with ComR. Unless it is the case that E. coli is equipped to process ComS to XIP or perhaps that ComS cleaves autocatalytically, our finding that E. coli carrying inducible *comR* and *comS* can activate a *comX* reporter plasmid is further evidence that processing of ComS to XIP is not absolutely required.

Other studies show some precedent for such regulation. Structural studies in S. pyogenes have shown that some intracellular Rgg receptor proteins can bind pheromones that differ in length and sequence (35). Crystallographic structures of homologous ComR proteins (34, 36) showed that the SHP binding pocket of the ComR C terminus falls in the tetratricopeptide repeat domain that is responsible for multimerization, while the N terminus helix-turn-helix structure binds DNA after an induced structural rearrangement. The location of the SHP binding pocket could allow the longer ComS to hinder multimerization when bound, resulting in a monomer binding to its target, while XIP does not. Figure S3 shows preliminary evidence that the ComS N terminus affects ComR binding in S. mutans. Neither ComS nor XIP could induce DNA binding by an N-terminally 6 histidine-tagged ComR, whereas XIP (but not ComS) caused DNA binding activity in a C-terminally tagged ComR. These data indicate that steric effects around the SHP binding pocket may influence DNA binding affinities.

Positive feedback occurs in many quorum sensing systems as the accumulation of the chemical signal in the extracellular environment stimulates the cell to produce additional signal or its cognate receptor. For example, in Vibrio fischeri the C8 homoserine lactone autoinducer stimulates expression of *ainS*, which encodes the autoinducer synthase (37). In Vibrio cholerae, the CAI-1 signal stimulates production of its CqsS receptor (38). In these cases, the extracellular signal concentration, which is the positive-feedback signal, is sensed by large numbers of cells, and so the population responds homogenously. However, if an individual cell responds preferentially to its own signal production, then the feedback signal is specific to the individual cell and the behavior is qualitatively different. Individual feedback can convert a graded (or unimodal) population response to a switched or bimodal response (39). Depending on parameters such as the rate of signal production and the level of noise or the cell density, the response of the cells may then span a range from strongly social or quorum behavior to purely autocrine or self-sensing (33) behavior in which cells respond independently and the population becomes heterogeneous (40). Synthetic biology has exploited this phenomenon in several bacterial quorum sensing systems to amplify the cell’s sensitivity to an exogenous signal. This can lower the quorum circuit’s threshold sensitivity to the signal, and it can also enhance the amplitude of the cell’s full response to that signal. Data in panels A and B of Fig. 1 suggest that the presence of the chromosomal *comS* gene in S. mutans roughly doubles the amplitude of the *comX* response and lowers the XIP sensitivity threshold roughly 2-fold. This amplification is comparable to what was accomplished in engineered synthetic systems (41, 42).

As a result, the ComRS system may have two modes of function in S. mutans. During early growth, CSP signaling stimulates the intracellular feedback behavior in ComRS, leading to population bimodality in *comX* expression levels. Consequently, only cells in a small subpopulation activate the late competence genes. However, in later growth phases or in mature biofilms, stress mechanisms that drive autolysis allow the release of XIP, generating a diffusing signal that is detected by other cells and amplified through the internal feedback mechanism, eliciting a broader competence response in the population. In this sense, XIP may serve to broadcast localized stress conditions, stimulating S. mutans to scavenge DNA resources opportunistically from nearby lysing cells (43, 44).

## MATERIALS AND METHODS

### Strains and growth conditions.

S. mutans wild-type strain UA159 and mutant reporting/gene deletion strains from glycerol freezer stock were grown in BBL BHI (Becton, Dickinson and Co.) at 37˚C in 5% CO_2_ overnight. The following antibiotics (concentrations) were used where resistance is indicated in Table 1: erythromycin (10 µg ml^−1^), kanamycin (1 mg ml^−1^), and spectinomycin (1 mg ml^−1^). For experiments performed in defined medium, strains were washed twice by centrifugation followed by removal of supernatant and resuspension in the defined medium FMC (45) (see Text S2 in the supplemental material for the ingredients of FMC). These were then diluted 20-fold into fresh FMC and allowed to grow under the same incubator conditions until an optical density at 600 nm (OD_600_) of 0.1 was reached. Synthetic XIP (sequence GLDWWSL) was synthesized and purified to 98% purity by NeoBioSci (Cambridge, MA).

**TABLE 1:** list of strains and plasmids used

Strain or plasmid	Characteristics^*a*^	Source or reference
S. mutans strains		
P*comX-gfp* (plasmid)	UA159 harboring P*comX* GFP promoter fusion on plasmid pDL278	11
P*comX-rfp*	UA159 harboring P*comX* dsRed RFP promoter fusion on plasmid pDL278	26
P*comX-gfp* Δ*comS*	UA159 *comS* gene replaced with a nonpolar erythromycin resistance cassette; harboring P*comX* GFP promoter fusion on plasmid pDL278	11
184*comS* P*comX-rfp*	UA159 harboring pIB184*comS* and P*comX* dsRed RFP promoter fusion	26
184*comS* P*comX-rfp* Δ*comS*	UA159 harboring pIB184*comS* and P*comX* dsRed RFP promoter fusion; *comS* disrupted by point mutation in start codon (ATG to AAG)	This study
E. coli strains		
BL21(DE3)	Used for recombinant protein expression	New England Biolabs, MA
10-beta	Used for propagating plasmids during cloning	New England Biolabs, MA
SAMU215	BL21(DE3) carrying P*comX-gfp* on pDL27	This study
SAMU216	SAMU215 carrying pACYC-Duet1 empty vector	This study
SAMU217	SAMU215 carrying pACYC-Duet1 plasmid with *comR* on multiple cloning site (MCS) I	This study
SAMU218	SAMU215 carrying pACYC-Duet1 plasmid with *comS* on MCS II	This study
SAMU219	SAMU215 carrying pACYC-Duet1 plasmid with *comR* on MCS I and *comS* on MCS II	This study
Plasmids		
pIB184	Shuttle expression plasmid with the P23 constitutive promoter, Em^r^	29
pDL278	E. coli-*Streptococcus* shuttle vector, Sp^r^	46
pET45b(+)his-*comR*_UA159_	pET45b(+) derivative containing the translational fusion P_T7lac_-*6xhis-comR*_UA159_, Ap^r^	This study
pACYC-Duet 1	T7-*lac* inducible expression vector for coexpression of two proteins, Cm^r^	Millipore-Sigma, MA

a Em^r^, erythromycin resistance; Sp^r^, spectinomycin resistance; Ap^r^, ampicillin resistance; Cm^r^, chloramphenicol resistance.

10.1128/mSphere.00444-18.2 TEXT S2 FMC ingredients. Download Text S2, PDF file, 0.5 MB. Copyright © 2018 Underhill et al. 2018 Underhill et al. This content is distributed under the terms of the Creative Commons Attribution 4.0 International license.

E. coli strains were grown in LB at 37°C with shaking in an aerobic incubator overnight. The following antibiotics (concentrations) were used where resistance is indicated: ampicillin (10 µg ml^−1^) and chloramphenicol (34 µg ml^−1^). For recombinant ComR expression experiments, the next day, the overnight cultures were diluted 100-fold into LB containing ampicillin at the indicated concentration and grown under the same incubator conditions as were used to grow the overnight cultures. For analysis of recombinant *comRS* expression inducing P*comX-gfp*, cells were diluted 100-fold into LB containing chloramphenicol and spectinomycin and grown with shaking at 37°C to an OD_600_ of 0.1, at which point IPTG was added and the cells were grown with shaking at 30°C until imaging was performed.

### Construction of *comS* point mutant.

The start codon of the *comS* gene was mutated from ATG to AAG. The mutation was introduced directly into the chromosome by site-directed mutagenesis using a PCR product generated by overlap extension PCR (47). Potential mutants were screened using mismatch amplification mutation analysis (MAMA) PCR (48), as previously described (49, 50). The point mutation was confirmed by PCR and sequencing to ensure that no further mutations were introduced into the *comS* gene and its flanking regions.

### Microfluidic experiments.

Microfluidic experiments were performed using a seven-channel polydimethylsiloxane (PDMS)-cast mixing array device, with fluorescence imaging and single-cell image analysis performed as described previously (11, 51, 52). Cells carrying a P*comX-gfp* plasmid-based reporter were grown to ab OD_600_ of 0.1 from dilution in FMC medium and were sonicated briefly using a Fisher Scientific FB120 sonic dismembrator probe to split large chains. Sonicated cells were then loaded into the device through a syringe capped with a 5-µm-pore-size filter to remove any remaining aggregations. FMC medium containing 1 mg ml^−1^ spectinomycin and a XIP gradient produced from three inlets containing different concentrations of XIP (0 nM, 600 nM, and 6 µM XIP inlets) passed through a mixing matrix was pumped through the cell chambers at a steady rate of 0.08 ml h^−1^ to create a constant, different XIP concentration in each cell chamber.

Gamma distributions (two-parameter probability sdistribution describing the amount of protein produced in sequential transcription and translation steps) were fitted to the single-cell fluorescence distributions using Matlab to fit protein production to theoretical descriptions (28). The fit was applied to cells fluorescing at levels above an arbitrary cutoff of 40 units (around the background level) in order to prevent turned-off cells from skewing the distribution. Parameter values were rounded to three significant figures, and data are reported in Table S1 in the supplemental material.

10.1128/mSphere.00444-18.6 TABLE S1 Parameters for gamma distribution fits to single-cell P*comX* GFP fluorescence distributions in microfluidic experiments. Download Table S1, DOCX file, 0.01 MB. Copyright © 2018 Underhill et al. 2018 Underhill et al. This content is distributed under the terms of the Creative Commons Attribution 4.0 International license.

10.1128/mSphere.00444-18.7 TABLE S2 Fitted values for the 12 parameters of the feedback activation model and statistical measurement of their robustness from bootstrap process. Download Table S2, DOCX file, 0.01 MB. Copyright © 2018 Underhill et al. 2018 Underhill et al. This content is distributed under the terms of the Creative Commons Attribution 4.0 International license.

### Flow rate dependence experiment.

In order to measure the flow rate dependence of XIP signaling, we loaded cells into a commercial six-channel microfluidic slide (IBIDI µ-slide VI; IBIDI GmbH). The six channels (channels a to f) contained respectively (channel a) a red fluorescent protein (dsRed) *comX* reporting strain (P*comX-rfp*) control channel flowing fresh BHI at 0.1 ml h^−1^; (channels b to e) four channels containing *comS* overexpression strain 184*comS* P*comX-rfp* (*comS* on plasmid pIB184 under the control of the strong constitutive P23 promoter) with BHI at different flow rates ranging from 0.02 ml h^−1^ to 1 ml h^−1^, and (channel f) a 184*comS* P*comX-rfp* strain with a point mutation disrupting the chromosomal *comS* gene (mutant Δ*comS*) under conditions of flow at 0.1 ml h^−1^. After 2 h, the neat BHI supplied was replaced with BHI supplemented with 50 µg ml^−1^ chloramphenicol in order to halt further translation and allow any RFP in the cells to fold. This was supplied at a flow rate of 0.1 ml h^−1^ for all channels. Four hours (the maturation time of our RFP) after chloramphenicol addition, final fluorescence images of the cultures were taken. Due to the bimodal *comX* activation in BHI, the fluorescence cutoff level was set as the maximum RFP fluorescence observed in the PcomX-*rfp* negative control. Cells exhibiting RFP fluorescence above this level were collected in an array, and the size of this sample as a percentage of the population and the median of the above-cutoff fluorescence are reported.

### Channel coculture experiment.

We loaded cocultures of a P*comX-gfp* Δ*comS* mutant (responders) with the *comS*-overexpressing strain 184*comS* P*comX-rfp* (senders) into two commercial microfluidic slides (IBIDI µ-slide VI) using static (not flowing) FMC medium and various ratios of *comS* overproducers/mutant Δ*comS* responders (percentage by volume of cultures at an OD_600_ of 0.1 subjected to vortex mixing together). Strains P*comX-rfp*, P*comX-gfp* Δ*comS*, P*comX-rfp* plus 50 nM XIP, and P*comX-gfp* Δ*comS* plus 50 nM XIP were used as controls. The end ports of the channels were sealed with mineral oil to prevent drying of the medium in the channels. Images were taken as described for the microfluidic experiments, and analysis was performed similarly. In the case of the controls, XIP was added to planktonic culture and the tube subjected to vortex mixing before pipetting into the slide. Because the population was heterogeneous with respect to both fluorescent reporter type and *comX* expression, a fluorescence threshold was defined as the maximum RFP fluorescence observed in the P*comX-rfp* negative control as described previously. The median of the RFP fluorescence observed above this cutoff level in other samples was used as a measure of how strongly the red cells were activating *comX* as a function of their number density.

### OD dependence of coculture response.

For tests of growth-phase dependence of signaling, cocultures similar to those in microfluidic channel slides were prepared. Overnight cultures were washed and diluted 40× into fresh FMC medium containing erythromycin (10 µg ml^−1^) and spectinomycin (1 mg ml^−1^). Once grown to an OD_600_ of 0.05, these were mixed in various ratios ranging from 0% *comS* overexpressers to 100% overexpressers, defined by the volume of *comS* overproducers added divided by the volume of the Δ*comS* culture added. Low initial cell densities were used to ensure that the early, middle, and late growth phases were probed for XIP release. Every 2 h, the OD_600_ of the culture and its pH were measured. The pH was corrected back to 7.0 using 2 N sodium hydroxide in cases in which it had deviated to below 6.5, in order to measure the reactions to any XIP released at late times into the culture. RFP fluorescence and GFP fluorescence were measured by pipetting a small amount of the culture onto a glass coverslip and analyzing single cells. GFP fluorescence at the 99th percentile was then used to determine if XIP was being released to the *comS* mutants in an OD_600_-dependent manner.

### RT-qPCR measurement of *comS*, *comR*, and *comX* transcripts.

S. mutans cells were diluted 20-fold into BHI medium (strain P*comX-gfp* wild type/BHI [WT/BHI], 184*comS PcomX-rfp*, and 184comS P*comX-rfp* Δ*comS* samples) or FMC medium (strain P*comX-gfp* WT/FMC ± XIP, P*comX-gfp* Δ*comS* ± XIP, pIB184/WT, and pIB184ComS/WT samples). Where added, XIP was supplied at OD_600_ = 0.1. Cells were harvested at OD_600_ = 0.5 by centrifugation and resuspended in RNA protectant buffer for 10 min. Samples were then centrifuged, the supernatant was removed, and the pellets were frozen at −80°C. RNA extraction was performed using a Qiagen RNEasy minikit (Qiagen, USA). RNA sample concentration and purity were measured using a Thermo Scientific NanoDrop One Microvolume UV-Vis spectrophotometer (Thermo Scientific, USA). RNA (1 µg) was then reverse transcribed to cDNA using a Bio-Rad iScript reverse transcription kit with random primers (Bio-Rad, USA). The qPCR was performed on a Bio-Rad CFX96 real-time system using Bio-Rad Sso Advanced Universal SYBR green Supermix with a 50-fold dilution of the cDNA and 500 nM gene-specific primers. Sequences used for the primers are given in Table S3. A standard curve across 8 orders of magnitude of transcript copies (from 10^8^ to 10^1^) was used to determine thetranscript count for each gene. For each sample the *comX*, *comR*, and *comS* transcript counts were then normalized by the 16S rRNA count for the same sample. Figure S1 in the supplemental material shows the median of this ratio, with error bar lengths given by the range from second-lowest to second-highest ratio obtained.

10.1128/mSphere.00444-18.8 TABLE S3 Primer sequences used for RT-qPCR. Download Table S3, DOCX file, 0.02 MB. Copyright © 2018 Underhill et al. 2018 Underhill et al. This content is distributed under the terms of the Creative Commons Attribution 4.0 International license.

### Fluorescence polarization.

ComR protein was obtained by cloning the *comR* gene into the 6×-His tagged site on pET-45b(+) vector in E. coli 10-beta using standard PCR cloning methods. His-ComR was then expressed in E. coli BL21(DE3) by induction with 1 mM IPTG at mid-exponential phase in LB. After 4 h, the cells were lysed using lysozyme in B-PER lysis buffer (Thermo Fisher). Protein was then purified from clarified lysate using nickel-nitrilotriacetic acid (Ni-NTA) agarose affinity chromatography and the histidine tag cleaved using EnterokinaseMax (EKMax) (Invitrogen) at 4°C. The resulting protein solution was dialyzed into phosphate-buffered saline (PBS) (pH 7.4) for experimental use. Native ComR concentrations were measured using the Pierce bicinchoninic acid (BCA) assay (Thermo Scientific), and the purity of the cleaved form was verified by SDS-PAGE run against an uncleaved sample.

Fluorescence polarization assays were performed in a 96-well plate with black bottom and black sides in a Biotek Synergy 2 plate reader (Biotek Instruments Inc.) in the polarization mode. A 5′ Bodipy FL-X-labeled self-annealing stem-loop DNA strand with sequence corresponding to PcomX (sequence 5′-Bodipy FL-X-ATGGGACATTTATGTCCTGTCCCCCACAGGACATAAATGTCCCAT-3′ [synthesized by Thermo Fisher]) was used as the binding aptamer, and a filter set (excitation wavelength, 485 nm; emission wavelength, 528 nm) was used for fluorescence excitation. Labeled DNA probe (1 nM) was added to a previously described reaction buffer (53) supplemented with 1 mM EDTA and 0.05 mg ml^−1^ salmon DNA. ComR was titrated in concentrations in this buffer alone or in the presence of 10 µM XIP or in the presence of 10 µM *comS*. The reaction mixtures were incubated at 37°C for 20 min before being read. Synthetic ComS (sequence, MFSILTSILMGLDWWSL) for use in fluorescence polarization was synthesized and purified to 60% purity by Biomatik (Wilmington, DE).

Competing unlabeled probe assays were performed with 1.5 µM ComR in the same buffer containing 1 nM P*comX* fluorescent DNA and 10 µM SHP (either ComS or XIP). An unlabeled probe corresponding to either the P*comS* probe (sequence, 5′-ACGGGACATAAATGTCCTGTCCCCCACAGGACATTTATGTCCCGT-3′; synthesized by Thermo Fisher) or the P*comX* probe described above was titrated into this solution and the decreasing polarization plotted. Reaction mixtures were again incubated at 37°C for 20 min before polarization readings were taken. In all FP experiments, reading was performed three times on the same plate to estimate instrument error. The average polarization was used for plotting and analysis. Details and parameters of the two-step binding model for the FP data are given in Text S3 in the supplemental material.

10.1128/mSphere.00444-18.2 TEXT S3 Methods for modeling of fluorescence polarization binding data of Fig. 5. Download Text S2, DOCX file, 0.01 MB. Copyright © 2018 Underhill et al. 2018 Underhill et al. This content is distributed under the terms of the Creative Commons Attribution 4.0 International license.

### ComRS expression in E. coli.

A recombinant *comRS* system was produced in E. coli by inserting *comR* into multiple cloning site I (MCS I) in pACYC-Duet 1 vector and *comS* into MCS II by standard cloning methods. BL21(DE3) cells containing P*comX-gfp* on pDL278 were transformed with the resulting pACYC-Duet 1 *comRS* plasmid. Controls consisting of empty pACYC-Duet 1 and the vector containing only *comR* at MCS I and only *comS* at MCS II were produced in the same way. Cells were diluted 100-fold from overnight cultures into LB containing chloramphenicol and spectinomycin and were grown with shaking at 37°C until they reached an OD_600_ of 0.1. At that point, IPTG was added in various amounts to induce expression of the T7-*lac* controlled recombinant system. Following addition of IPTG, the cells were grown with shaking at 30°C and GFP fluorescence was imaged after 3 h.

### Mathematical model of *comRS* control of *comX*.

Deterministic modeling of *comX* activation by *comRS* was performed by least-squares fitting of a chemical equilibrium model to the microfluidic data from the experiments performed for each of the wild-type background P*comX* GFP strain and the Δ*comS* cells. Details of the model and the robustness analysis are given in the supplemental material, with parameter values given in Table S2.
