# Erratum for Underhill et al., “Intracellular Signaling by the *comRS* System in Streptococcus mutans Genetic Competence”

**DOI:** 10.1128/mSphere.00042-19

**Published:** 2019-02-13

**Authors:** Simon A. M. Underhill, Robert C. Shields, Justin R. Kaspar, Momin Haider, Robert A. Burne, Stephen J. Hagen

**Affiliations:** a Department of Physics, University of Florida, Gainesville, Florida, USA; b Department of Oral Biology, University of Florida, Gainesville, Florida, USA

## ERRATUM

Volume 3, no. 5, e00444-18, 2018, https://doi.org/10.1128/mSphere.00444-18. In the originally published Fig. 1, the labels for panels C and D contain some incorrect values for XIP concentrations. The correct concentrations are given in the revised portion of the figure shown below.

**Figure 1:**
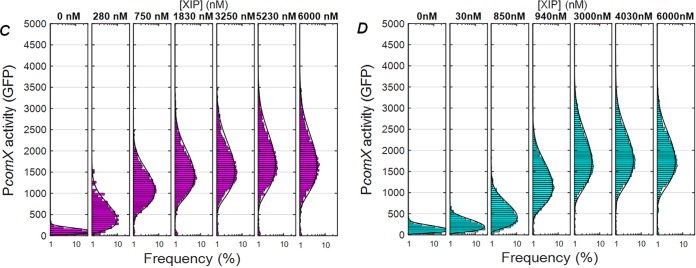


In the “Construction of *comS* point mutant” paragraph of the Materials and Methods section, the following sentence should be added at the end. “The *comS* point mutant is an unpublished strain that was constructed and kindly provided by Sang-Joon Ahn of the University of Florida College of Dentistry.”

