# Supplementary figures and images for: Intracellular Signaling by the comRS System in Streptococcus mutans Genetic Competence
Source: mSphere. 2018 Oct 31;3(5):e00444-18. doi: 10.1128/mSphere.00444-18 (PMC6211226; doi:10.1128/mSphere.00444-18)

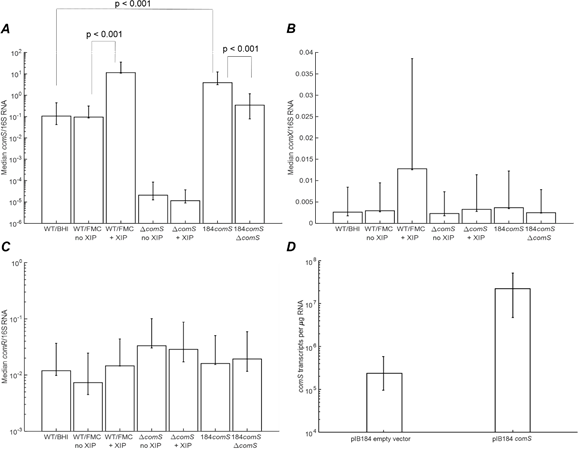

Supplement: FIG S1 [file sph006182682sf1.tif]

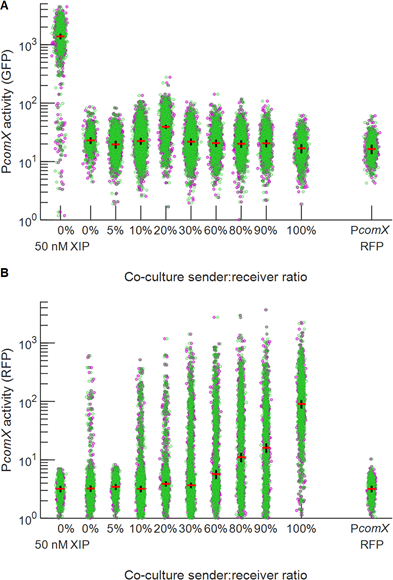

Supplement: FIG S2 [file sph006182682sf2.tif]

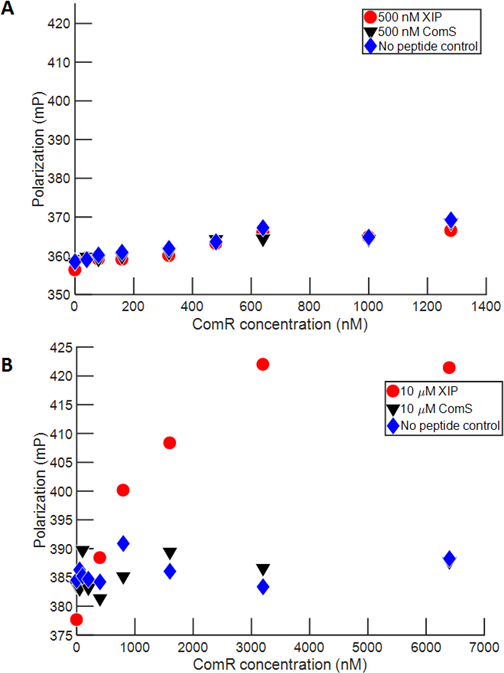

Supplement: FIG S3 [file sph006182682sf3.tif]
