# Supplementary material for: Intracellular Signaling by the comRS System in Streptococcus mutans Genetic Competence
Source: mSphere. 2018 Oct 31;3(5):e00444-18. doi: 10.1128/mSphere.00444-18 (PMC6211226; doi:10.1128/mSphere.00444-18)
Supplement: TEXT S1 [file sph006182682s1.docx]

**Deterministic fit: Equation system and calculated parameter values**

A mathematical model (below) was constructed for the ComRS activation of *comX* with intracellular feedback, in the presence of extracellular XIP, and this model was used to fit the *comX* activation data in Fig. 1. The same offset and multiplicative factor were used to map calculated [ComX] onto the GFP fluorescence curves for both strains, as this is a property of the GFP, not the gene circuit. To model the *comS-*deficient strain, parameters representing *comS* feedback and constitutive production were set to zero. ComR was assumed to be present at around 15 copies per cell, as only modest changes in its expression result from early competence inducing factors (54). Exogenous XIP was taken to be a non-depleting reservoir. The system of ODEs used to fit microfluidic data is given below. X represents ComX, Z the internal XIP concentration, S the internal ComS concentration, R the (constant) ComR concentration and Exo the exogenous XIP level. All units are in nM and seconds where appropriate. Other symbols are parameters describing the reaction kinetics. A star indicates one of the *V* parameters contributing to feedback, while unstarred *V*s indicate a maximum rate of production of ComX. Hill kinetics corresponding to inferred cooperativity from FP assays were used. Calculated parameters are given in Table S2. A 200-iteration bootstrap analysis of the data was performed in order to estimate parameter robustness, with the 10^th^ and 90^th^ percentiles of parameter values reported. These percentile values demonstrate preservation of the relative order of magnitude between dissociation constants for XIP-ComR and ComS-ComR.

$\frac{dX}{dt}= \frac{V_{1}R^{2}Z^{2}}{R^{2}Z^{2}+ K_{X}^{4}}+ \frac{V_{2}RS}{RS+ K_{S}^{2}}- \beta_{X}X$ (1)

$\frac{dS}{dt}= \alpha_{0}- \beta_{S}S- \gamma S+ \frac{V_{1}^{*}R^{2}Z^{2}}{R^{2}Z^{2}+ K_{X}^{4}}+ \frac{V_{2}^{*}RS}{RS+ K_{S}^{2}}- \frac{V_{2}RS}{RS+ K_{S}^{2}}$ (2)

$\frac{dZ}{dt}=J\left( Exo-Z \right)- \beta_{Z}Z+ \gamma S- \frac{V_{1}R^{2}Z^{2}}{R^{2}Z^{2}+ K_{X}^{4}}- \frac{V_{1}^{*}R^{2}Z^{2}}{R^{2}Z^{2}+ K_{X}^{4}}$ (3)

**Robustness analysis for model**

Robustness of fit was tested through the bootstrap method, using the 90th and 10th percentile behavior of parameters to examine whether the transcriptional efficiency difference hypothesized was preserved in this range. Dependence on the initial parameter guess was checked by 50 iterations of adding a Gaussian-distributed random number with a mean of the best fit parameter and standard deviation half the best fit parameter to the start guess vector components used to find the best fit. New sets of fit parameters for each of these were then generated. It was found that the ComS-ComR complex elicited higher *comX* transcription in 100% of cases than did the XIP-ComR complex, and higher *comS* feedback stimulation (V* parameters) in 78% of cases. Thus while numerous solutions to the system exist, the *comX* transcriptional efficiency discrepancy hypothesized is a generic property of the fit.
