# Supplementary material for: Intracellular Signaling by the comRS System in Streptococcus mutans Genetic Competence
Source: mSphere. 2018 Oct 31;3(5):e00444-18. doi: 10.1128/mSphere.00444-18 (PMC6211226; doi:10.1128/mSphere.00444-18)
Supplement: TEXT S2 [file sph006182682s2.pdf]

FMC Growth Medium

Component	***Stock Solution Concn (g/100 ml)***	***Volume (ml) of Stock Soln per 100 ml FMC***
**1** $KHPO_4$		1
KH_2PO_4	8.84	
K_2HPO_4	6.1	
**2** $(NH_4)_2SO_4$	12	1
**3** Amino Acid Mix		4
L-Aspartic Acid	0.5	
L-Phenylalanine	0.5	
L-Serine	0.5	
L-Proline	1	
L-Hydroxyproline	1	
Glycine	1	
**4** L-Leucine	0.5	4
**5** L-Glutamic Acid	3	2
**6** DL-Alanine	2	2
**7** L-Isoleucine	1	2
**8** L-Methionine	1	2
**9** L-Threonine	2	1
**10** L-Arginine	2	2
**11** L-Histidine	2	2
**12** L-Tryptophan	2	2
**13** L-Valine	2	1
**14** L-Lysine	2	1
**15** Riboflavin	0.008	1
**16** Vitamin Mix		1
4-aminobenzoic acid	0.0016	
Thiamine-HCl	0.008	
Nicotinamide	0.04	
**17** Pantothenate	0.0172	1
**18** Biotin	0.005	0.2
**19** Folic Acid	0.002	1
**20** Pyridoxal	0.023	1
**21** L-Glutamine	0.1	1
**22** L-Cystine	1	4
**23** L-Tyrosine	1	4
**24** A-G-U		6
Adenine sulfate-$2H_2O$	0.174	
Guanine HCl	0.124	
Uracil	0.1	
**25** Salts B		1
$MgSO_4$-$7H_2O$	4	
NaCl	0.2	
$FeSO_4$-$7H_2O$	0.2	
$MnSO_4$-H_2O	0.2	
**26** Sodium Acetate (in grams)		0.6
**27** Sodium Citrate (in grams)		0.225
**28** Sodium Carbonate (in grams)		0.136
**29** Water		50
