# Supplementary material for: Intracellular Signaling by the comRS System in Streptococcus mutans Genetic Competence
Source: mSphere. 2018 Oct 31;3(5):e00444-18. doi: 10.1128/mSphere.00444-18 (PMC6211226; doi:10.1128/mSphere.00444-18)
Supplement: TABLE S1 [file sph006182682st1.docx]

**Table S1: Parameters for gamma distribution fits to single cell P*comX* GFP fluorescence distributions in microfluidic experiments**.

| [XIP] | *a* | *b* |
| --- | --- | --- |
| **Wild type** | - | - |
| 0 | 4.09 | 5.51 |
| 280 | 5.50 | 172 |
| 700 | 9.62 | 153 |
| 1840 | 11.0 | 184 |
| 3250 | 12.5 | 168 |
| 5230 | 12.4 | 174 |
| 6000 | 11.7 | 172 |
| **Δ*comS*** | - | - |
| 0 | 6.14 | 2.29 |
| 30 | 7.10 | 2.44 |
| 850 | 4.24 | 166 |
| 940 | 5.11 | 148 |
| 3000 | 7.44 | 160 |
| 4020 | 7.61 | 172 |
| 6000 | 7.45 | 180 |
