# Supplementary material for: Intracellular Signaling by the comRS System in Streptococcus mutans Genetic Competence
Source: mSphere. 2018 Oct 31;3(5):e00444-18. doi: 10.1128/mSphere.00444-18 (PMC6211226; doi:10.1128/mSphere.00444-18)
Supplement: TABLE S2 [file sph006182682st2.docx]

**Table S2: Fitted values for the 12 parameters of the model and statistical measurement of their robustness from bootstrap process.**

| Parameter | Best fit value – used for Fig. 7 | 10^th^ percentile from bootstrap | 90^th^ percentile from bootstrap | Units |
| --- | --- | --- | --- | --- |
| $\alpha_{0}$ | 7.85 | 1.78 | 19.1 | nM s^-1^ |
| $\beta_{S}$ | 7.17 x 10^-3^ | 7.97 x 10^-4^ | 1.32 x 10^-2^ | s^-1^ |
| $\gamma$ | 0.452 | 0.227 | 2.74 | s^-1^ |
| $V_{1}^{*}$ | 1.07 x 10^4^ | 3.8 x 10^3^ | 2.46 x 10^4^ | nM s^-1^ |
| $V_{2}^{*}$ | 1.33 x 10^4^ | 3.22 x 10^3^ | 2.65 x 10^4^ | nM s^-1^ |
| $K_{x}$ | 148 | 42 | 210 | nM |
| $K_{S}$ | 2740 | 849 | 3000 | nM |
| $V_{1}$ | 3.55 | 0.988 | 558 | nM s^-1^ |
| $V_{2}$ | 778 | 309 | 2190 | s^-1^ |
| $J$ | 9.39 | 6.14 x 10^-2^ | 17.6 | s^-1^ |
| $\beta_{Z}$ | 1.28 | 0.264 | 3.07 | nM s^-1^ |
| $\beta_{X}$ | 10.5 | 1.71 | 16.2 | s^-1^ |
