# Supplementary material for: Intracellular Signaling by the comRS System in Streptococcus mutans Genetic Competence
Source: mSphere. 2018 Oct 31;3(5):e00444-18. doi: 10.1128/mSphere.00444-18 (PMC6211226; doi:10.1128/mSphere.00444-18)
Supplement: TABLE S3 [file sph006182682st3.docx]

**Table S3: RT-qPCR primer sequences**

| Gene and direction | Primer sequence |
| --- | --- |
| *comX* forward | 5’-CGTCAGCAAGAAAGTCAGAAA C-3’ |
| *comX* reverse | 5’-ATACCGCCACTTGACAAACAG-3’ |
| *comS* forward | 5’-TCAAAAAGAAAGGAGAATAACA-3’ |
| *comS* reverse | 5’-TCATCTGAGATAAGGGCTGT-3’ |
| *comR* forward | 5’-TATTACGAAGGCCAACCTAT-3’ |
| *comR* reverse | 5’-TTCTTCTTCAGGCAAATGAT-3’ |
| 16S rRNA forward | 5’-CACACCGCCCGTCACACC-3’ |
| 16S rRNA reverse | 5’-CAGCCGCACCTTCCGATACG-3’ |
