# Supplementary material for: Intracellular Signaling by the comRS System in Streptococcus mutans Genetic Competence
Source: mSphere. 2018 Oct 31;3(5):e00444-18. doi: 10.1128/mSphere.00444-18 (PMC6211226; doi:10.1128/mSphere.00444-18)
Supplement: TEXT S3 [file sph006182682s3.docx]

**Modeling the fluorescence polarization binding data**

We compared the FP data to a two-step binding model in which the peptide ComS or XIP forms a multimeric complex with ComR (with dissociation constant k_1_), and then a single copy of this complex binds to the fluorescent DNA probe (with dissociation constant k_2_), increasing its fluorescence anisotropy. The model is summarized by

P + R ⇌ C C + D ⇌ D* C + U ⇌ U*

Here *P* is the peptide (ComS or XIP), *R* is ComR, *C* is the peptide-ComR multimeric complex, *D* (*U*) is the free labeled (unlabeled) probe, *D** (*U**) is the labeled (unlabeled) probe with complex bound. The order of multimerization of the complex *C* is *n*. We solved the equilibrium equations for the model using the multivariate Newton-Raphson method in Matlab. We performed separate data analyses for the FP data ComS and XIP, respectively. In each analysis we searched for parameter values (*k_1_*, *k_2_*, *n*) that simultaneously minimized the sum of squares residuals for both the association (Figure 5A) and competition (Figure 5B) experiments for a given peptide *P*.

In general the FP data are compatible with a range of parameter values. If *n* is constrained to be less than 2.5 then optimal values are in the range *k_1_* ∼ 1-6 μM and *k_2_* ∼ 1-30 nM and *n* ≃ 2-2.5 for for XIP interacting with ComR, and *k_1_* ∼ 3-20 μM and *k_2_* ∼ 30-200 nM and *n* ≃ 1.6-2.5 for ComS interacting with ComR.
